# Subtyping patients with somatic tinnitus: Modulation of tinnitus and history for somatic dysfunction help identify tinnitus patients with temporomandibular joint disorders

**DOI:** 10.1371/journal.pone.0202050

**Published:** 2018-08-13

**Authors:** Massimo Ralli, Antonio Greco, Armando Boccassini, Giancarlo Altissimi, Carlo Di Paolo, Vincenzo Falasca, Armando De Virgilio, Antonella Polimeni, Giancarlo Cianfrone, Marco de Vincentiis

**Affiliations:** 1 Department of Oral and Maxillofacial Sciences, Sapienza University of Rome, Rome, Italy; 2 Center for Hearing and Deafness, University at Buffalo, Buffalo, New York, United States of America; 3 Department of Sense Organs, Sapienza University of Rome, Rome, Italy; 4 Otorhinolaryngology Unit, Humanitas Clinical and Research Center, Rozzano, MI, Italy; University of Regensburg, GERMANY

## Abstract

**Objective:**

Determine in a cohort of patients with normal hearing and chronic tinnitus if self-reported history for temporomandibular joint (TMJ) dysfunction and a positive modulation of tinnitus in the TMJ region could be suggestive of an underlying TMJ disorder.

**Patients and methods:**

The study included 226 patients presenting to the Head and Neck Service of our University Hospital. Following audiological and somatic tinnitus evaluation, patients were divided into two groups. The study group (n = 134) included subjects that met both the following criteria: A) a self-reported history for TMJ dysfunction and B) a positive modulation of tinnitus following somatic maneuvers in the TMJ region. The control group (n = 92) included patients with similar demographic and tinnitus characteristics that did not meet the proposed criteria for somatic tinnitus. Afterwards, patients underwent clinical TMJ evaluation in the Service of Clinical Gnathology of our University.

**Results:**

One hundred thirty-one patients (57.9%) received a clinical diagnosis of TMJ disorder according to DC/TMD Axis I; 79.1% in the study group and 27.2% in the control group. Ninety-five (42.1%) patients were negative for TMJ disorders; 20.9% in the study group and 72.8% in the control group. A significantly higher number of TMJ disorders was found in patients in the study group compared to the control group (p<0.0001). Most patients had joint disorders (67.2%), followed by other (29.8%) and pain disorders (29%). Logistic regression analysis in the study group showed that female gender was more prevalent in patients with TMJ disorders.

**Conclusion:**

Our findings in patients with chronic tinnitus and normal hearing suggest that self-reported history for somatic dysfunction and modulation of tinnitus, when occurring simultaneously in the TMJ region, can be useful to preliminarily identify patients with TMJ disorders.

## Introduction

Tinnitus is defined as the perception of sound without an accompanying external auditory stimulus. Tinnitus can follow hearing loss, ototoxicity, and psychiatric comorbidity [1–9]. In a portion of patients, tinnitus can be associated to somatic disorders, often in the absence of hearing loss; this is defined as “somatic tinnitus” [10–12]. The most common conditions in somatic tinnitus are temporomandibular joint (TMJ) and head and neck disorders [10, 12–22]. Somatic tinnitus is often underdiagnosed [23, 24].

Patients with somatic tinnitus can benefit from specific treatments of the associated somatic disorder [25–32]; however, it is still unclear from the literature if there are specific characteristics that could help selecting patients with somatic tinnitus [15, 19, 23, 24, 33, 34].

Tinnitus modulation by movements of the head and neck, limbs and eyes following interactions between the auditory and the somatosensory systems has been proposed as a possible indicator for somatic tinnitus [11, 12, 17–19, 35, 36]. Tinnitus modulation has been largely reported in different patient series with an incidence ranging between 65.3% and 83.3% [14–16, 18, 24, 37]; however, although modulation appears to be increased in patients with somatic tinnitus [17, 20, 24–26, 38, 39], the capability to modulate tinnitus itself may not indicate the presence of an underlying somatic disorder and therefore should not be used as the sole indicator for the somatic origin of tinnitus [19].

In a recent study from our group, we proposed that the correlation between a positive self-reported history for a somatic dysfunction and positive tinnitus modulation in the same region could be suggestive of a somatic disorder underlying tinnitus [13]. The aim of this study is to further investigate this hypothesis evaluating the presence of clinically diagnosed TMJ disorders in tinnitus patients with a self-reported history for TMJ dysfunction and a positive modulation of tinnitus in the TMJ region (study group) compared with patients not matching these criteria (control group).

## Materials and methods

### Participants

This study was conducted in 226 patients with normal hearing and chronic tinnitus recruited among those presenting to the Head and Neck Service of our University Hospital (Policlinico Umberto I, Sapienza University Rome, Italy) between January 2016 and June 2017 with tinnitus as their chief compliant.

Clinically normal hearing was defined as an individual hearing threshold ≤25 dB HL at frequencies from 250 to 4,000 Hz at the octave scale in both ears according to the American Academy of Otolaryngology and American Council of Otolaryngology [40]. Chronic tinnitus was defined as continuous tinnitus lasting for more than 12 months at the time of the examination.

Exclusion criteria were pulsatile tinnitus, history of acoustic trauma, middle or inner-ear disease (e.g., otosclerosis, chronic suppurative otitis media or endolymphatic hydrops), significant interaural hearing asymmetry, retrocochlear disease, previous ear surgery, concurrent medical treatment for tinnitus except for antioxidant drugs.

All patients underwent audiological and somatic tinnitus evaluation in the Tinnitus Unit of the Department of Sense Organs. Following audiological and somatic evaluation, patients were divided into two groups. The study group (n = 134) included subjects that met both the following criteria: A) a self-reported history for TMJ dysfunction and B) a positive modulation of tinnitus following somatic maneuvers in the TMJ region. The control group (n = 92) included patients with similar demographic (age, gender) and tinnitus (length, side) characteristics that did not meet the proposed criteria for somatic tinnitus. Afterwards, patients underwent clinical TMJ evaluation in the Service of Clinical Gnathology of the Department of Oral and Maxillofacial Sciences.

Patients signed a written informed consent; the procedures performed were in accordance with the ethical standards of the ethics committee on human experimentation of the Department of Sense Organs of the Sapienza University of Rome, that specifically approved this study, and with the Helsinki Declaration.

### Audiological evaluation

Patients underwent anamnestic evaluation, a full otolaryngology examination and audiological test battery including pure tone audiometry (PTA) and acoustic immittance test. PTA was measured at frequencies of 0.125, 0.25, 0.50, 0.75, 1, 2, 3, 4, 6, and 8 kHz; hearing was considered symmetrical if thresholds for each ear occurred within 10dB of each other. Tinnitus side, pitch, and loudness were tracked for each patient; characteristics included tinnitus side (unilateral or bilateral) and tinnitus description from a predefined set of possibilities including “buzzing”, “whistle”, “high-pitched”, “low-pitched” and “other”. All subjects were asked to complete the Italian versions of the Tinnitus Handicap Inventory (THI) [41], Hearing Handicap Inventory (HHI) [42], the Hyperacusis Questionnaire (HQ) [43], and the Geräuschüberempfindlichkeit Questionnaire (GUF) [44].

### Somatic tinnitus evaluation

Somatic tinnitus evaluation included anamnestic investigation of self-reported history for TMJ dysfunction and assessment of tinnitus modulation.

Self-reported history for TMJ dysfunction was considered positive if the patient reported one or more of the following events occurring before the onset of tinnitus: 1) head trauma involving TMJ region; 2) intensive manipulation of teeth or jaw; 3) recurrent pain episodes in the TMJ region; 4) increase of both TMJ pain and tinnitus at the same time; and 5) intense periods of bruxism during day or night [23].

Tinnitus modulation assessment was performed as previously described [13, 15]. Five somatic TMJ maneuvers were performed to elicit changes in tinnitus loudness modulation (increase/decrease). Patients were asked to perform a specific movement or to resist pressure applied by the examiner against the jaw. Each contraction was held for 10 seconds. Maneuvers were performed in the same order for each patient. If the assessment resulted in positive tinnitus modulation, the examiner waited for tinnitus to return to baseline levels before proceeding with another maneuver. Tinnitus modulation was considered present when the patient reported tinnitus modulation following at least one somatic maneuver. Maneuvers used for somatic testing in the present study are detailed in Table 1.

**Table 1:** Maneuvers used for somatic testing in our study.

Jaw Maneuvers	
TMJ 1	Clench teeth together
TMJ 2	Open the mouth with restorative pressure
TMJ 3	Protrude jaw with restorative pressure
TMJ 4	Slide jaw to left with restorative pressure
TMJ 5	Slide jaw to right with restorative pressure

Temporomandibular joint (TMJ) maneuvers performed to elicit changes in tinnitus loudness modulation.

### Temporomandibular joint evaluation

TMJ was evaluated by a specialized dentist according to Diagnostic Criteria for Temporomandibular Disorders Axis I (DC/TMD), the most commonly used diagnostic criteria for TMJ disorder evaluation characterized by simple, reliable, and valid operational definitions for the history, examination, and imaging procedures needed to render physical diagnoses in both clinical and research settings [45]. Patients were studied using clinical, anamnestic, and instrumental protocols to evaluate the presence and the stage of their dysfunctional pathology and/or the presence of any structural deterioration of osteoarticular and muscular components, fulfilling the diagnostic research criteria for TMJ disorders. Physical examination of the TMJ and head and neck muscles was performed. Diagnostic imaging included orthopanoramic and skull radiographs in all patients.

### Statistical analysis

Statistical analyses were performed using Prism GraphPad v7. Descriptive statistics, mean, and standard deviation were calculated for numeric variables; frequency and percentages were calculated for categorical variables. Unpaired t test was used to evaluate differences between patients in the two groups for numeric variables. Chi-square test of association was performed to assess association between two categorical variables. Multiple logistic regression was performed to identify the variables associated with TMJ disorders. The p-value for assessing statistical significance was an alpha of 0.05.

## Results

### Demographic and audiological characteristics

**Fig 1:**
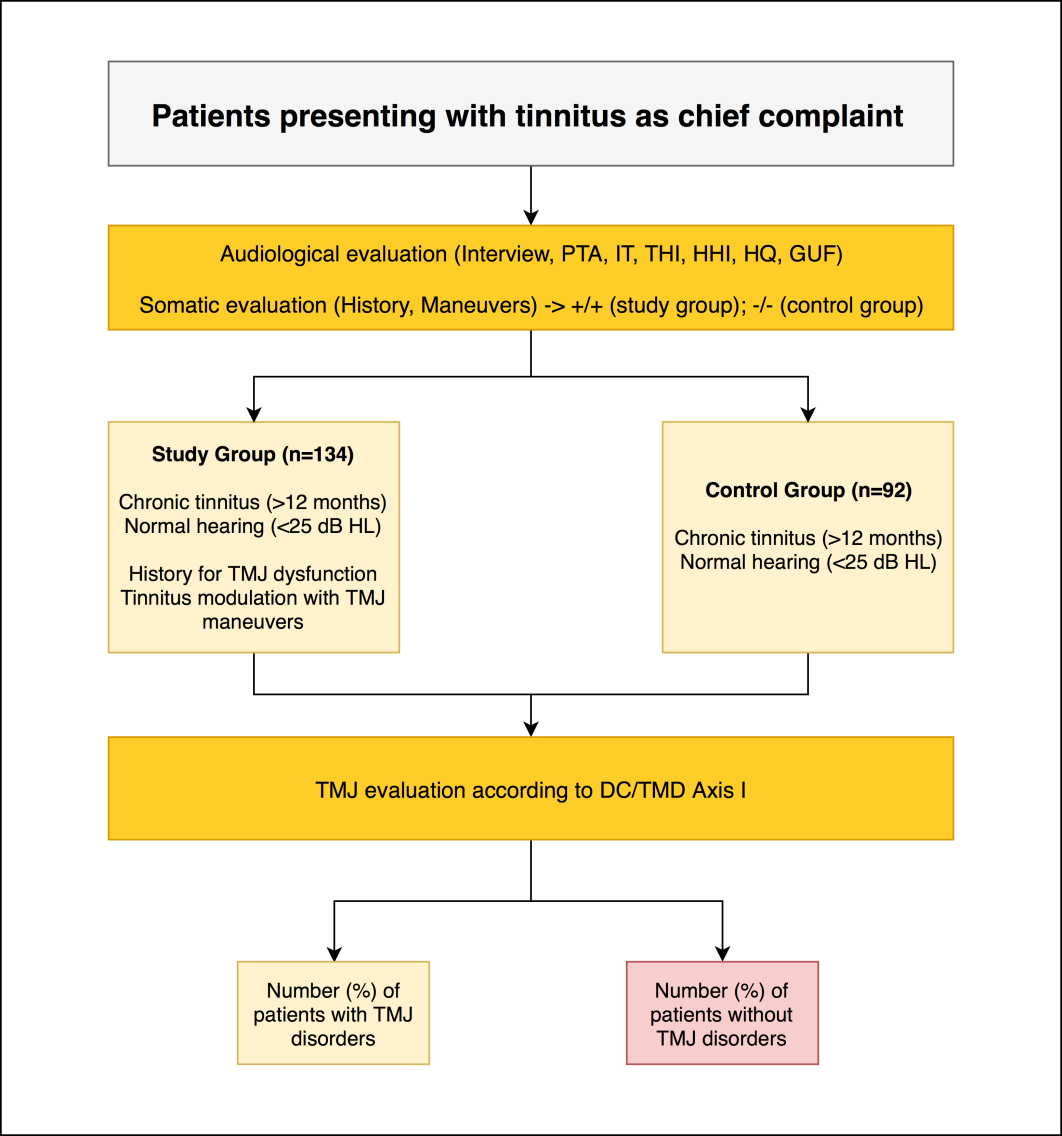
Study workflow for the present study. A total of 226 patients with clinically normal hearing and chronic tinnitus were enrolled in the study and underwent audiological and somatic evaluation. Patients were divided into a study group (n = 134) and a control group (n = 92) based on self-reported history of temporomandibular (TMJ) dysfunction and positive somatic modulation of tinnitus following somatic maneuvers in the TMJ region. Afterwards, all patients underwent gnathological evaluation to assess the presence of clinically diagnosed TMJ disorders according to Diagnostic Criteria for Temporomandibular Disorders (DC/TMD) Axis I. PTA: Pure Tone Audiometry; IT: Immittance Test; THI: Tinnitus Handicap Inventory; HHI: Hearing Handicap Inventory; HQ: Hyperacusis Questionnaire; GUF: Geräuschüberempfindlichkeit Questionnaire.

Two hundred twenty-six patients were enrolled in the study; 134 were included in the study group and 92 in the control group. One hundred twenty-eight patients were males (56.6%) and 98 were females (43.4%). Study workflow is shown in Fig 1.

In the study group, 78 (58.2%) were males, and 56 (41.8%) were females. Mean age was 48.5 years (range: 15–79 years, SD = 14.1). Average PTA thresholds were 16.8 dB HL (0.125–2 kHz), 20.4 dB HL (2–4 kHz), and 26.6 (4–8 kHz) with no significant interaural asymmetries. The average duration of tinnitus at the time of the first admission was 4.9 years (SD = 6.6). Tinnitus was bilateral in 75 patients (55.9%) and unilateral in 59 (44.1%). Tinnitus was described by patients as “high-pitch” in 43 patients (32.1%), “low-pitch” in 35 (26.1%), “whistle” in 32 (23.9%), “buzzing” in 14 (10.4%), and “other” in 10 (7.5%). Mean THI score was 46.9 (SD = 20.8), mean HHI was 15.1 (SD = 17.1), mean HQ was 14.2 (SD = 7.8) and mean GUF score was 10.2 (SD = 7.2).

In the control group, 50 (54.3%) were males, and 42 (45.7%) were females. Mean age was 45.9 years (range: 19–84 years, SD = 13.6). Average PTA thresholds were 14.9 dB HL (0.125–2 kHz), 19.8 dB HL (2–4 kHz), and 27.1 (4–8 kHz) with no significant interaural asymmetries. The average duration of tinnitus at the time of the first admission was 4.5 years (SD = 5.6). Tinnitus was bilateral in 51.1% of cases and unilateral in 48.9%. Tinnitus was described as “high-pitch” in 10.9%, “low-pitch” in 16.3%, “whistle” in 36.9%, “buzzing” in 26.1%, and “other” in 9.8% of cases. Mean THI score was 27.5 (SD = 20.1), mean HHI was 10.1 (SD = 7.3), mean HQ was 13.1 (SD = 10.6) and mean GUF score was 8.6 (SD = 6.7).

No significant differences between groups were found for age (p = 0.1639), gender (p = 0.5670), tinnitus length (p = 0.6225), laterality (p = 0.4715), HQ (p = 0.4017) and GUF (p = 0.0992). A significant difference was found for the THI (p<0.0001) and HHI (p = 0.0084) questionnaire scores. Demographic and tinnitus characteristics and questionnaire results are presented in *Table 2*.

**Table 2:** Demographic and audiological characteristics of patients in the study and control groups.

	Study group [Frequency (%)]	Control group [Frequency (%)]	p-value
Number [%]	134 (59.3)	92 (40.7)	
Age [mean, SD, range]	48.5, 14.1, 15–79	45.9, 13.6, 19–84	0.1639
Sex [Frequency (%)]			0.5670
Male	78 (58.2)	50 (54.3)	
Female	56 (41.8)	42 (45.7)	
Tinnitus length [Mean, SD, range]	4.9, 6.6, 1–40	4.5, 5.6, 1–28	0.6225
Tinnitus side [Frequency (%)]			0.4715
Bilateral	75 (55.9)	47 (51.1)	
Unilateral (left)	38 (28.3)	32 (34.8)	
Unilateral (right)	21 (15.8)	13 (14.1)	
Tinnitus sound [Frequency (%)]			
Buzzing	14 (10.4)	24 (26.1)	
High-Pitch	43 (32.1)	10 (10.9)	
Low-Pitch	35 (26.1)	15 (16.3)	
Whistle	32 (23.9)	34 (36.9)	
Other	10 (7.5)	9 (9.8)	
Questionnaires [mean, SD, range]			
THI	46.9, 20.8, 4–90	27.5, 20.1, 2–94	<0.0001 *
HHI	15.1, 17.1, 0–72	10.1, 7.3, 0–30	0.0084 *
HQ	14.2, 7.8, 0–35	13.1, 10.6, 0–30	0.4017
GUF	10.2, 7.2, 0–35	8.6, 6.7, 0–28	0.0992

Details of demographic and audiological characteristics and self-administered questionnaire scores of patients in the study and control groups.

* Indicates statistical significance.

### Clinical diagnosis of temporomandibular joint disorders

**Fig 2:**
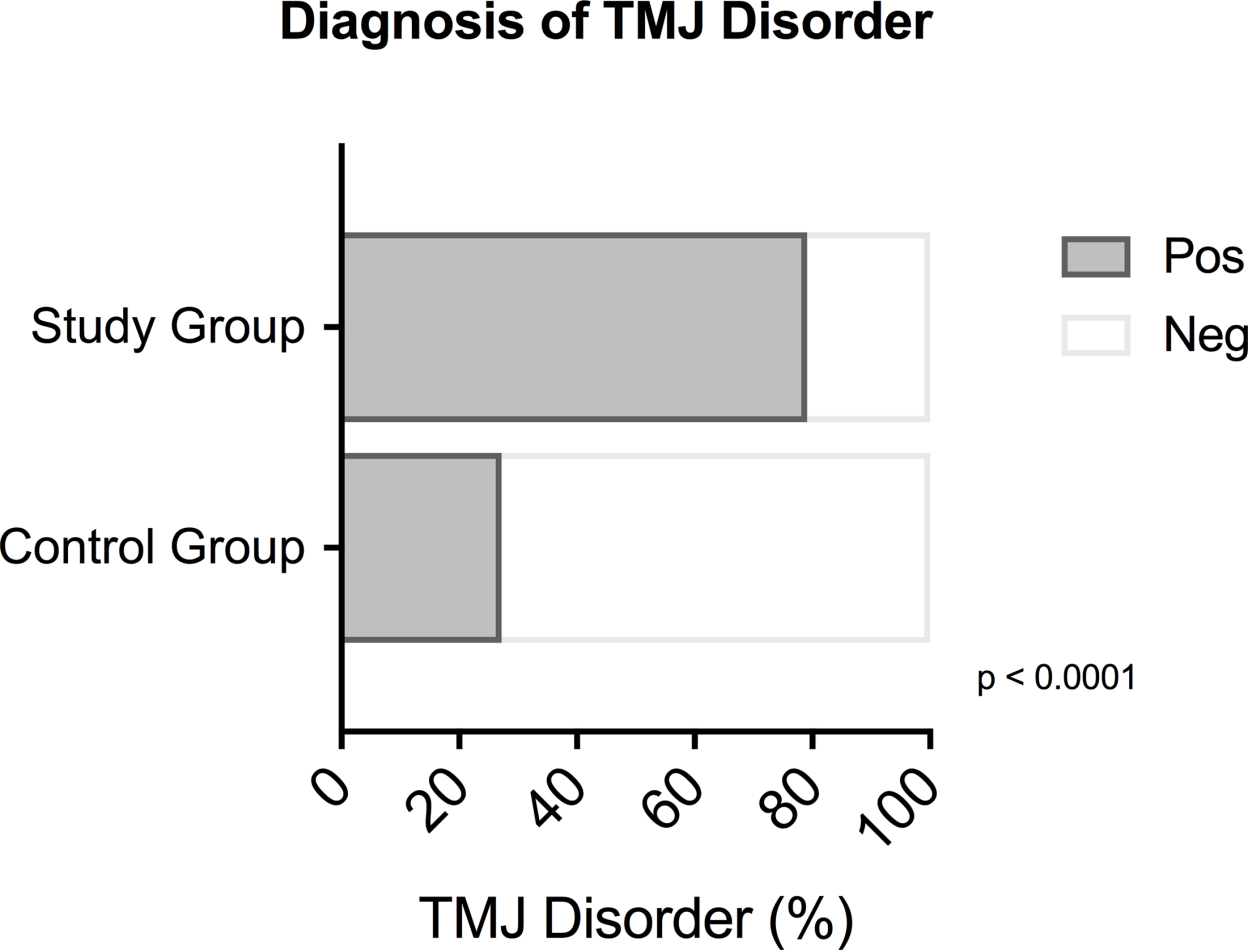
Diagnosis of temporomandibular joint (TMJ) disorders. Diagnosis of TMJ disorders according to Diagnostic Criteria for Temporomandibular Disorders (DC/TMD) Axis I in patients in the study and control groups. A significantly higher number of TMJ disorders was found in patients in the study group compared to the control group (p<0.0001).

One hundred thirty-one patients (57.9%) received a clinical diagnosis of TMJ disorder according to DC/TMD Axis I; 106/131 (79.1%) were in the study group and 25/131 (27.2%) in the control group. Ninety-five (42.1%) patients were negative for TMJ disorders; 28/95 (20.9%) in the study group and 67/95 (72.8%) in the control group. The difference between the study and control groups was statistically significant (p<0.0001) (Fig 2).

Among patients with a TMJ disorder diagnosis, 69 (52.7%) were males and 62 (47.3%) were females. Mean age was 47.4 years (range: 15–84 years, SD = 14.5). The average duration of tinnitus was 5.2 years (SD = 6.9); tinnitus was bilateral in 53.4% of cases and unilateral in 46.6%. Mean THI score was 43.9 (SD = 22.1), HHI was 14.3 (SD = 15.3), HQ was 14.3 (SD = 8.4) and mean GUF score was 10.2 (SD = 7.1).

Among patients without TMJ disorders, 59 (62.1%) were males and 36 (37.9%) were females. Mean age was 47.5 years (range: 19–74 years, SD = 13.1). The average duration of tinnitus was 4.3 years (SD = 5.4); tinnitus was bilateral in 54.7% of cases and unilateral in 45.3%. Mean THI score was 32.3 (SD = 21.6), HHI was 11.4 (SD = 12.3), HQ was 12.9 (SD = 9.8) and GUF was 8.5 (SD = 6.7).

No significant differences were found between patients with and without a diagnosis of TMJ disorder for age (p = 0.9432), gender (p = 0.0929), tinnitus length (p = 0.2524), laterality (p = 0.6443), HHI (p = 0.1295), HQ (p = 0.2431) and GUF (p = 0.0625). A significant difference was found only for the THI (p = 0.0001) questionnaire.

When comparing patients with and without TMJ disorders within the study and control groups, a higher prevalence of the female gender was found in patients with a diagnosis of TMJ disorders in the study group (48.1% vs 17.8%) (p = 0.0037), while no significant differences for age (p = 0.4360), tinnitus length (p = 0.9629), and laterality (p = 0.8893) were found. Similarly, self-administered questionnaire scores did not differ. No significant differences for demographic and tinnitus characteristics and questionnaire scores were found between patients with and without TMJ disorders in the control group. Detailed results are presented in Table 3.

**Table 3:** Characteristics of patients in the study and control groups with and without temporomandibular joint disorders.

	STUDY GROUP			CONTROL GROUP		
	Patients with TMJ disorders [Frequency (%)]	Patients without TMJ disorders [Frequency (%)]	p-value	Patients with TMJ disorders [Frequency (%)]	Patients without TMJ disorders [Frequency (%)]	p-value
Number [%]	106 (79.1)	28 (20.9)		25 (27.2)	67 (72.8)	
Age [mean, SD, range]	48.1, 14.3, 15–79	50.4, 13.1, 21–72	0.4360	44.7, 15.4, 23–84	46.3, 13, 19–74	0.6089
Sex [Frequency (%)]			0.0037			0.7853
Male	55 (51.9)	23 (82.1)		14 (56)	36 (53.7)	
Female	51 (48.1)	5 (17.9)		11 (44)	31 (46.3)	
Tinnitus length [Mean, SD, range	4.9, 6.8, 1–40	5, 5.7, 1–23	0.9629	6.7, 7.3, 1–28	4.1, 5.3, 1–25	0.0586
Tinnitus side [Frequency (%)]			0.8893			
Bilateral	59 (55.7)	16 (57.1)		11 (44)	36 (53.7)	
Unilateral (left)	29 (27.3)	9 (32.1)		8 (32)	23 (34.4)	
Unilateral (right)	18 (17)	3 (10.8)		6 (24)	8 (11.9)	
Tinnitus sound [Frequency (%)]						
Buzzing	11 (10.4)	2 (7.1)		5 (20)	20 (29.8)	
High-Pitch	30 (28.3)	13 (46.4)		4 (16)	7 (10.4)	
Low-Pitch	31 (29.2)	4 (14.3)		2 (8)	13 (19.4)	
Whistle	23 (21.7)	9 (32.1)		11 (44)	21 (31.3)	
Other	10 (9.4)	0 (-)		3 (12)	6 (8.9)	
Questionnaires [mean, SD, range]						
THI	48.1, 20.7, 12–90	42.4, 20.8, 4–84	0.1901	26.1, 18.8, 2–74	28, 20.7, 2–94	0.6751
HHI	14.8, 16.5, 0–72	16.5, 19.1, 0–64	0.7148	11.9, 7.5, 0–30	9.4, 7.2, 0–30	0.1401
HQ	14.4, 7.9, 1–37	13.3, 7.1, 0–27	0.5233	14.2, 10, 0–28	12.7, 10.8, 0–30	0.5712
GUF	10.6, 7.3, 0–35	8.8, 6.9, 0–26	0.2645	8.9, 6.6, 0–22	8.3, 6.7, 0–28	0.7120

Comparison of demographic and tinnitus characteristics and self-administered questionnaire scores in patients in the study and control groups with and without temporomandibular joint (TMJ) disorders.

**Fig 3:**
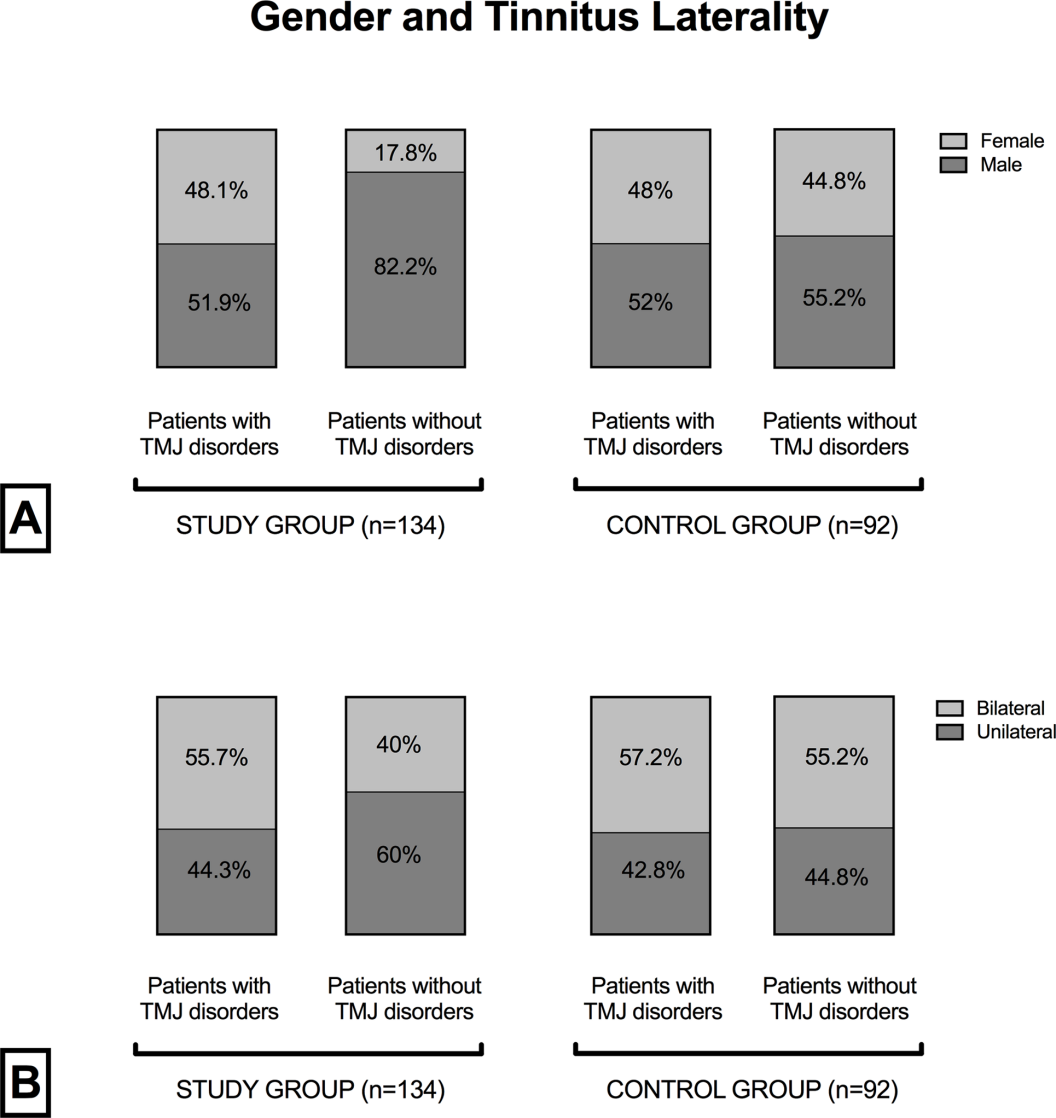
Relative proportions for gender and tinnitus laterality within groups. Comparison of relative proportion of (A) gender and (B) tinnitus laterality in dependence from a diagnosis of temporomandibular joint (TMJ) disorder in the study and control groups.

Relative proportions for gender and tinnitus laterality within groups are shown in Fig 3.

Logistic regression analysis was performed in the study group to investigate demographic characteristics, tinnitus length, and questionnaire variables associated with a positive diagnosis of TMJ disorder; analysis indicated that male gender was 0.21 times less common in patients with TMJ disorders than in patients without (p = 0.01, CI 0.07–0.65). No differences were seen for age, questionnaire score and tinnitus length (Table 4).

**Table 4:** Logistic regression analysis in the study group.

	Odds Ratio	p-value	CI Lower	CI Upper
Age	0.98	0.18	0.94	1.01
**Male**	**0.21**	**0.01**	**0.07**	**0.65**
Tinnitus length	1.03	0.42	0.96	1.11
THI	1.02	0.30	0.98	1.05
HHI	0.97	0.09	0.93	1.01
HQ	0.99	0.88	0.91	1.08
GUF	1.04	0.51	0.93	1.16

Logistic regression analysis for demographic characteristics, tinnitus length and questionnaire variables associated with a positive diagnosis of temporomandibular joint disorder. Significant results are shown in bold.

### Characteristics of temporomandibular joint disorders according to DC/TMD classification

**Fig 4:**
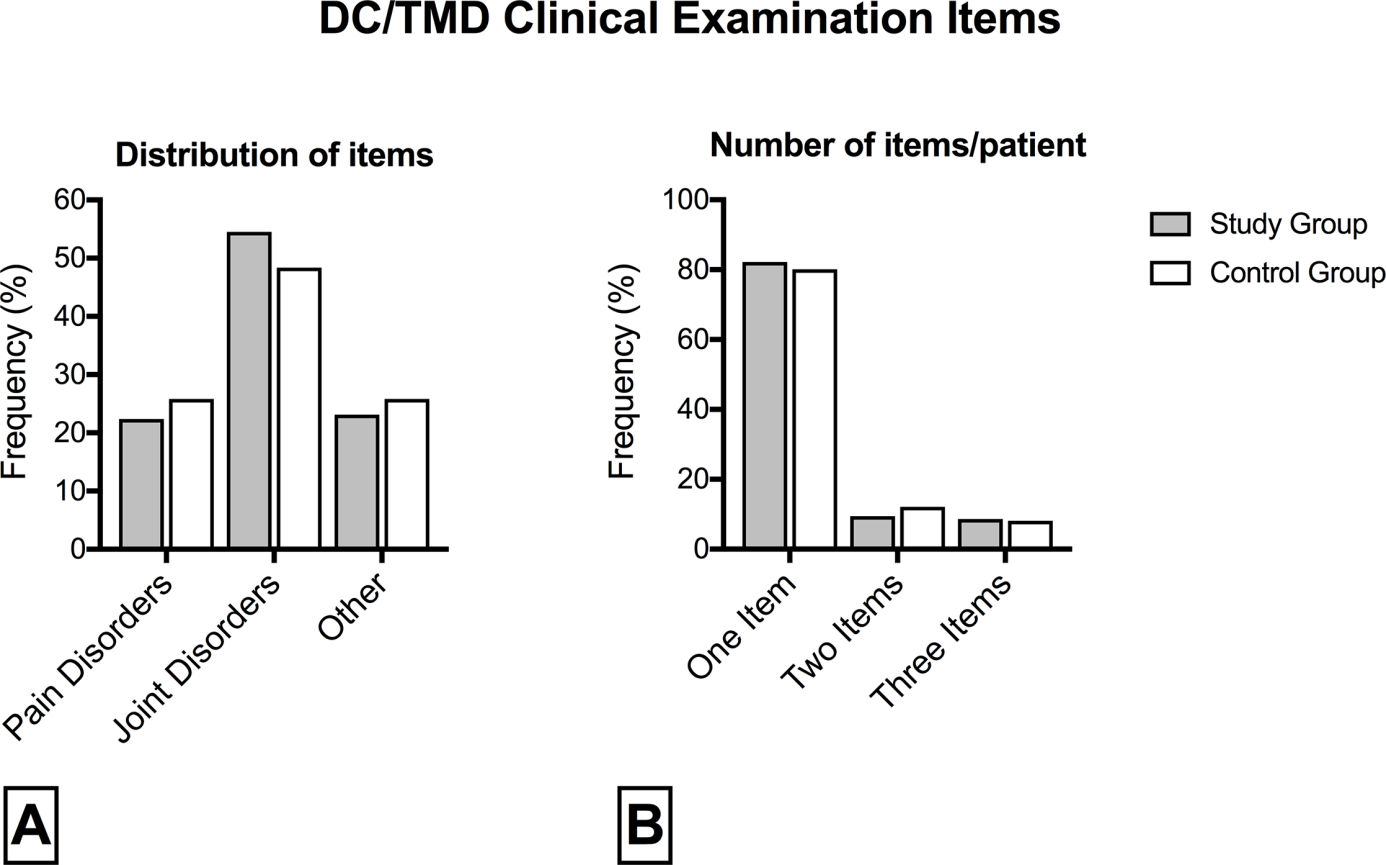
Temporomandibular joint (TMJ) disorders classification according to Diagnostic Criteria for Temporomandibular Disorders (DC/TMD) Axis I. (A) Distribution of items: most patients had joint disorders (67.2%), followed by other (29.8%) and pain disorders (29%). (B) Number of items per patient: most patients (81.6%) had only one clinical examination item; two or three concomitant items were found in 18.4% of patients. No significant differences were found between patients in the study and control groups.

TMJ disorders were classified according to DC/TMD Axis I classification. Results are shown in Fig 4.

Among patients with TMJ disorders, 88 patients (67.2%) had a diagnosis of joint disorder (disc displacement with/without reduction; degenerative joint disease; subluxation), 38 (29%) of pain disorder (myalgia; myofascial pain; arthralgia; headache attributed to TMJ disorders) and 39 (29.8%) were classified as “other” (malocclusion; parafunctional habits). One hundred seven patients (81.6%) had only one clinical examination item (joint OR pain OR other), 13 (9.9%) had two concomitant items (joint and pain disorders in 6 cases, joint and other disorders in 5 cases and pain and other disorders in 5 cases) and 11 (8.5%) had all three items. No significant differences were found between patients in the study and control groups for TMJ disorder type and number of concomitant clinical examination items.

## Discussion

### Clinical diagnosis of temporomandibular joint disorders in our sample

The present study evaluated the presence of TMJ disorders in tinnitus patients with normal hearing matching the criteria proposed by the authors for somatic tinnitus compared to a sample of patients with similar demographic and tinnitus characteristics that did not match such criteria. TMJ disorders were diagnosed according to standardized diagnostic criteria. A significantly larger number of patients in the study group received a clinical diagnosis of TMJ disorder compared to the control group. These results suggest that history and modulation of tinnitus, when occurring simultaneously in the TMJ region, may have a role for the preliminary selection of tinnitus patients that are more likely to have a TMJ somatic disorder, although an association between somatic disorders and pure somatic origin of tinnitus in these patients cannot be confirmed by the present data. Our findings are also supported by the prevalent theories in the current literature [20, 23, 24, 26, 27, 37].

### Characteristics of patients with temporomandibular joint disorders

About 70% of the patients with a clinical diagnosis of TMJ disorder had joint disorders, followed by pain disorders and other disorders of the TMJ. This is consistent with Buergers, who reported that most patients in their sample had joint disorders [27]. Most of the participants had only one clinical examination item, while about 18% exhibited two or more concomitant items. This differs from results presented by Buergers, who found that about 40% of his tinnitus patients had a DC/TMD diagnosis in more than one clinical examination item [25], and Fernandes [46, 47], who reported that tinnitus patients most likely had pain and joint disorders combined.

Logistic regression analysis indicated that female gender was more prevalent in patients with TMJ disorders than in patients without TMJ disorders in the study group; this is consistent with results previously reported by other authors that indicated a higher prevalence of female gender in patients with TMJ disorders [20, 26, 48].

No significant differences were found for tinnitus characteristics, such as pitch and duration, between patients with and without a diagnosis of TMJ disorder. This is consistent with previous findings from Wright et al and Vernon et al [27, 49].

### Clinical considerations

The association of tinnitus with somatic disorders has been reported by many authors [26, 28, 37, 38, 47, 50–53]; significant improvements in tinnitus have been described upon somatic treatment in patients with somatic tinnitus [25–32]. A comparison of 16 studies published between 1964 and 2016 on tinnitus changes following TMJ therapy showed that, on average, 69% of patients reported tinnitus improvement or complete resolution after TMJ disorder treatment, while 32% reported no changes [10]. De Felicio [29] reported significant improvements in tinnitus symptoms in patients with TMJ disorders using bite splints for eight weeks; Tullberg [30] reported that 2 years after TMJ disorder treatment with oral splints, 43% of subjects in the treatment group reported a decrease in tinnitus compared to 12% of subjects in the control group. Wright and Bifano [27] reported improvements in tinnitus symptoms in patients who had undergone cognitive therapy, bite splints and home exercises for the treatment of TMJ disorders. Buergers [25] reported improvement of tinnitus after stomatognathic treatment in 11 of 25 participants (44%). Other studies have shown higher percentages of improvement or complete remission of TMJ disorder-related tinnitus after various stomatognathic treatments, ranging from 43% to 86% [31, 32].

The present study shows that a larger number of patients in the study group compared to the control group received a clinical diagnosis of TMJ disorder; however, a direct correlation between the TMJ disorder and tinnitus can be only speculative. Future studies on the effects of specific TMJ therapy in patients selected according to these criteria are necessary to confirm the direct relationship between tinnitus and the underlying TMJ disorder in these patients.

### Limits of the study

This study has some limits. The effects of TMJ disorder treatment on tinnitus have not been investigated, as the present study was limited to the diagnosis of TMJ somatic disorders in patients matching the proposed criteria. Although tinnitus improvements in patients with somatic tinnitus have been extensively described after treatment of TMJ disorders by many authors [20, 25–32], a direct correlation between TMJ somatic disorders and tinnitus cannot be proved in enrolled patients with the current study design and further studies are necessary to confirm the validity of the proposed criteria for selection of somatic tinnitus patients.

Hidden and high-frequency hearing loss were not studied in our patients; audiological evaluation investigated frequencies up to 8 kHz and was not extended to higher frequencies. Given the spread of hidden hearing loss among the general population, and especially among tinnitus sufferers [54, 55], the presence of unexplored hidden hearing loss, especially in the 10–16 kHz range, should be considered, and could have played a role in tinnitus onset in our patients.

## Conclusions

The significantly higher number of clinically diagnosed TMJ disorders in patients with chronic tinnitus and normal hearing matching the proposed criteria compared to subjects in the control group suggests that self-reported history for somatic dysfunction and modulation of tinnitus, when occurring simultaneously in the TMJ region, can be useful to preliminarily identify patients with TMJ disorders. Given the elevate correlation between somatic disorders and tinnitus reported in the literature, such criteria should always be investigated when approaching tinnitus patients, as they could be relevant for specific tinnitus patient subtyping.

## References

[1] Baguley D, McFerran D, Hall D. Tinnitus. Lancet. 2013;382(9904):1600–7. 10.1016/S0140-6736(13)60142-7 . 23827090

[2] Ralli M, Balla MP, Greco A, Altissimi G, Ricci P, Turchetta R, et al Work-Related Noise Exposure in a Cohort of Patients with Chronic Tinnitus: Analysis of Demographic and Audiological Characteristics. Int J Environ Res Public Health. 2017;14(9):E1035 10.3390/ijerph14091035 . 28885581 PMC5615572

[3] Folmer RL, Griest SE, Meikle MB, Martin WH. Tinnitus severity, loudness, and depression. Otolaryngol Head Neck Surg. 1999;121(1):48–51. 10.1016/S0194-5998(99)70123-3 . 10388877

[4] Heller AJ. Classification and epidemiology of tinnitus. Otolaryngol Clin North Am. 2003;36(2):239–48. . 12856294 10.1016/s0030-6665(02)00160-3

[5] Langguth B, Kreuzer PM, Kleinjung T, De Ridder D. Tinnitus: causes and clinical management. Lancet Neurol. 2013;12(9):920–30. 10.1016/S1474-4422(13)70160-1 . 23948178

[6] Moller AR. Epidemiology of Tinnitus in Adults. Textbook of Tinnitus. 2011:29–37

[7] Ralli M, Altissimi G, Di Stadio A, Mazzei F, Turchetta R, Cianfrone G. Relationship between hearing function and myasthenia gravis: A contemporary review. J Int Med Res. 2017;45(5):1459–65. Epub 2016/11/12. 10.1177/0300060516672124 ; PubMed Central PMCID: PMCPMC5718710. 27834304 PMC5718710

[8] Ralli M, Troiani D, Podda MV, Paciello F, Eramo SL, de Corso E, et al The effect of the NMDA channel blocker memantine on salicylate-induced tinnitus in rats. Acta Otorhinolaryngol Ital. 2014;34(3):198–204. Epub 2014/06/03. 24882929; PubMed Central PMCID: PMCPMC4035835. PMC4035835 24882929

[9] Sheppard A, Hayes SH, Chen GD, Ralli M, Salvi R. Review of salicylate-induced hearing loss, neurotoxicity, tinnitus and neuropathophysiology. Acta Otorhinolaryngol Ital. 2014;34(2):79–93. Epub 2014/05/21. ; PubMed Central PMCID: PMCPMC4025186. 24843217 PMC4025186

[10] Ralli M, Greco A, Turchetta R, Altissimi G, de Vincentiis M, Cianfrone G. Somatosensory tinnitus: Current evidence and future perspectives. J Int Med Res. 2017;45(3):933–47. Epub 2017/05/30. 10.1177/0300060517707673 ; PubMed Central PMCID: PMCPMC5536427. 28553764 PMC5536427

[11] Shore S, Zhou J, Koehler S. Neural mechanisms underlying somatic tinnitus. Prog Brain Res. 2007;166:107–23. 10.1016/S0079-6123(07)66010-5 ; PubMed Central PMCID: PMCPMC2566901. 17956776 PMC2566901

[12] Wu C, Stefanescu RA, Martel DT, Shore SE. Tinnitus: Maladaptive auditory-somatosensory plasticity. Hear Res. 2016;334:20–9. 10.1016/j.heares.2015.06.005 ; PubMed Central PMCID: PMCPMC4676957. 26074307 PMC4676957

[13] Ralli M, Altissimi G, Turchetta R, Mazzei F, Salviati M, Cianfrone F, et al Somatosensory Tinnitus: Correlation between Cranio-Cervico-Mandibular Disorder History and Somatic Modulation. Audiol Neurootol. 2016;21(6):372–82. Epub 2017/01/19. 10.1159/000452472 . 28099967

[14] Simmons R, Dambra C, Lobarinas E, Stocking C, Salvi R. Head, Neck, and Eye Movements That Modulate Tinnitus. Semin Hear. 2008;29(4):361–70. 10.1055/s-0028-1095895 ; PubMed Central PMCID: PMCPMC2633109. 19183705 PMC2633109

[15] Won JY, Yoo S, Lee SK, Choi HK, Yakunina N, Le Q, et al Prevalence and factors associated with neck and jaw muscle modulation of tinnitus. Audiol Neurootol. 2013;18(4):261–73. Epub 2013/07/25. 10.1159/000351685 . 23881235

[16] Abel MD, Levine RA. Muscle contractions and auditory perception in tinnitus patients and nonclinical subjects. Cranio. 2004;22(3):181–91. Epub 2004/08/06. 10.1179/crn.2004.024 . 15293775

[17] Levine RA. Somatic (craniocervical) tinnitus and the dorsal cochlear nucleus hypothesis. Am J Otolaryngol. 1999;20(6):351–62. . 10609479 10.1016/s0196-0709(99)90074-1

[18] Levine RA, Abel M, Cheng H. CNS somatosensory-auditory interactions elicit or modulate tinnitus. Exp Brain Res. 2003;153(4):643–8. 10.1007/s00221-003-1747-3 . 14600798

[19] Levine RA, Nam EC, Oron Y, Melcher JR. Evidence for a tinnitus subgroup responsive to somatosensory based treatment modalities. Prog Brain Res. 2007;166:195–207. 10.1016/S0079-6123(07)66017-8 . 17956783

[20] Vielsmeier V, Strutz J, Kleinjung T, Schecklmann M, Kreuzer PM, Landgrebe M, et al Temporomandibular joint disorder complaints in tinnitus: further hints for a putative tinnitus subtype. PLoS One. 2012;7(6):e38887 Epub 2012/06/23. 10.1371/journal.pone.0038887 ; PubMed Central PMCID: PMCPMC3378537. 22723902 PMC3378537

[21] Ward J, Vella C, Hoare DJ, Hall DA. Subtyping Somatic Tinnitus: A Cross-Sectional UK Cohort Study of Demographic, Clinical and Audiological Characteristics. PLoS One. 2015;10(5):e0126254 Epub 2015/05/23. 10.1371/journal.pone.0126254 ; PubMed Central PMCID: PMCPMC4440784. 25996779 PMC4440784

[22] Bhatt J, Ghavami Y, Lin HW, Djalilian H. Cervical Spine Dysfunctions in Patients with Chronic Subjective Tinnitus. Otol Neurotol. 2015;36(8):1459–60. Epub 2015/07/25. 10.1097/MAO.0000000000000827 . 26208127

[23] Sanchez TG, Rocha CB. Diagnosis and management of somatosensory tinnitus: review article. Clinics (Sao Paulo). 2011;66(6):1089–94. 10.1590/S1807-59322011000600028 ; PubMed Central PMCID: PMCPMC3129953. 21808880 PMC3129953

[24] Sanchez TG, da Silva Lima A, Brandao AL, Lorenzi MC, Bento RF. Somatic modulation of tinnitus: test reliability and results after repetitive muscle contraction training. Ann Otol Rhinol Laryngol. 2007;116(1):30–5. Epub 2007/02/20. 10.1177/000348940711600106 . 17305275

[25] Buergers R, Kleinjung T, Behr M, Vielsmeier V. Is there a link between tinnitus and temporomandibular disorders? J Prosthet Dent. 2014;111(3):222–7. 10.1016/j.prosdent.2013.10.001 . 24286640

[26] Vielsmeier V, Kleinjung T, Strutz J, Burgers R, Kreuzer PM, Langguth B. Tinnitus with temporomandibular joint disorders: a specific entity of tinnitus patients? Otolaryngol Head Neck Surg. 2011;145(5):748–52. Epub 2011/06/28. 10.1177/0194599811413376 . 21705788

[27] Wright EF, Bifano SL. The Relationship between Tinnitus and Temporomandibular Disorder (TMD) Therapy. Int Tinnitus J. 1997;3(1):55–61. 10753366

[28] Rubinstein B, Axelsson A, Carlsson GE. Prevalence of signs and symptoms of craniomandibular disorders in tinnitus patients. J Craniomandib Disord. 1990;4(3):186–92. 2098394

[29] de Felicio CM, Melchior Mde O, Ferreira CL, Da Silva MA. Otologic symptoms of temporomandibular disorder and effect of orofacial myofunctional therapy. Cranio. 2008;26(2):118–25. Epub 2008/05/13. 10.1179/crn.2008.016 . 18468271

[30] Tullberg M, Ernberg M. Long-term effect on tinnitus by treatment of temporomandibular disorders: a two-year follow-up by questionnaire. Acta Odontol Scand. 2006;64(2):89–96. Epub 2006/03/21. 10.1080/00016350500377842 . 16546850

[31] Bush FM. Tinnitus and otalgia in temporomandibular disorders. J Prosthet Dent. 1987;58(4):495–8. Epub 1987/10/01. . 3478483 10.1016/0022-3913(87)90282-4

[32] Rubinstein B, Carlsson GE. Effects of stomatognathic treatment on tinnitus: a retrospective study. Cranio. 1987;5(3):254–9. Epub 1987/07/01. . 3476212 10.1080/08869634.1987.11678198

[33] Haider HF, Hoare DJ, Costa RFP, Potgieter I, Kikidis D, Lapira A, et al Pathophysiology, Diagnosis and Treatment of Somatosensory Tinnitus: A Scoping Review. Front Neurosci. 2017;11:207 Epub 2017/05/16. 10.3389/fnins.2017.00207 ; PubMed Central PMCID: PMCPMC5408030. 28503129 PMC5408030

[34] Ralli M, Salvi RJ, Greco A, Turchetta R, De Virgilio A, Altissimi G, et al Characteristics of somatic tinnitus patients with and without hyperacusis. PLoS One. 2017;12(11):e0188255 Epub 2017/11/22. 10.1371/journal.pone.0188255 ; PubMed Central PMCID: PMCPMC5697853. 29161302 PMC5697853

[35] Shore SE. Plasticity of somatosensory inputs to the cochlear nucleus—implications for tinnitus. Hear Res. 2011;281(1–2):38–46. 10.1016/j.heares.2011.05.001 ; PubMed Central PMCID: PMCPMC3174344. 21620940 PMC3174344

[36] Cacace AT. Expanding the biological basis of tinnitus: crossmodal origins and the role of neuroplasticity. Hear Res. 2003;175(1–2):112–32. . 12527130 10.1016/s0378-5955(02)00717-7

[37] Sanchez TG, Guerra GC, Lorenzi MC, Brandao AL, Bento RF. The influence of voluntary muscle contractions upon the onset and modulation of tinnitus. Audiol Neurootol. 2002;7(6):370–5. 10.1159/000066155 . 12401968

[38] Saldanha AD, Hilgenberg PB, Pinto LM, Conti PC. Are temporomandibular disorders and tinnitus associated? Cranio. 2012;30(3):166–71. 10.1179/crn.2012.026 . 22916668

[39] Tuz HH, Onder EM, Kisnisci RS. Prevalence of otologic complaints in patients with temporomandibular disorder. Am J Orthod Dentofacial Orthop. 2003;123(6):620–3. Epub 2003/06/14. 10.1016/S0889540603001537 . 12806339

[40] AAO-ACO. Guide for the evaluation of hearing handicap. JAMA. 1979;241(19):2055–9. . 430800

[41] Passi S, Ralli G, Capparelli E, Mammone A, Scacciatelli D, Cianfrone G. The THI questionnaire: psychometric data for reliability and validity of the Italian version. Int Tinnitus J. 2008;14(1):26–33. . 18616083

[42] Ventry IM, Weinstein BE. The hearing handicap inventory for the elderly: a new tool. Ear Hear. 1982;3(3):128–34. . 7095321 10.1097/00003446-198205000-00006

[43] Khalfa S, Dubal S, Veuillet E, Perez-Diaz F, Jouvent R, Collet L. Psychometric normalization of a hyperacusis questionnaire. ORL J Otorhinolaryngol Relat Spec. 2002;64(6):436–42. 10.1159/000067570 . 12499770

[44] Nelting M, Rienhoff NK, Hesse G, Lamparter U. [The assessment of subjective distress related to hyperacusis with a self-rating questionnaire on hypersensitivity to sound]. Laryngorhinootologie. 2002;81(5):327–34. 10.1055/s-2002-28342 . 12001021

[45] Schiffman E, Ohrbach R, Truelove E, Look J, Anderson G, Goulet JP, et al Diagnostic Criteria for Temporomandibular Disorders (DC/TMD) for Clinical and Research Applications: recommendations of the International RDC/TMD Consortium Network* and Orofacial Pain Special Interest Groupdagger. J Oral Facial Pain Headache. 2014;28(1):6–27. Epub 2014/02/01. 10.11607/jop.1151 ; PubMed Central PMCID: PMCPMC4478082. 24482784 PMC4478082

[46] Fernandes G, Franco AL, Goncalves DA, Speciali JG, Bigal ME, Camparis CM. Temporomandibular disorders, sleep bruxism, and primary headaches are mutually associated. J Orofac Pain. 2013;27(1):14–20. Epub 2013/02/21. 10.11607/jop.921 . 23424716

[47] Fernandes G, Goncalves DA, de Siqueira JT, Camparis CM. Painful temporomandibular disorders, self reported tinnitus, and depression are highly associated. Arq Neuropsiquiatr. 2013;71(12):943–7. 10.1590/0004-282X20130191 . 24347013

[48] Stouffer JL, Tyler RS. Characterization of tinnitus by tinnitus patients. J Speech Hear Disord. 1990;55(3):439–53. Epub 1990/08/01. . 2381186 10.1044/jshd.5503.439

[49] Vernon J, Griest S, Press L. Attributes of tinnitus associated with the temporomandibular joint syndrome. Eur Arch Otorhinolaryngol. 1992;249(2):93–4. Epub 1992/01/01. . 1581053 10.1007/BF00186455

[50] Bernhardt O, Gesch D, Schwahn C, Bitter K, Mundt T, Mack F, et al Signs of temporomandibular disorders in tinnitus patients and in a population-based group of volunteers: results of the Study of Health in Pomerania. J Oral Rehabil. 2004;31(4):311–9. 10.1046/j.1365-2842.2003.01249.x . 15089935

[51] Ferendiuk E, Zajdel K, Pihut M. Incidence of otolaryngological symptoms in patients with temporomandibular joint dysfunctions. Biomed Res Int. 2014;2014:824684 10.1155/2014/824684 ; PubMed Central PMCID: PMCPMC4094732. 25050373 PMC4094732

[52] Lee CF, Lin MC, Lin HT, Lin CL, Wang TC, Kao CH. Increased risk of tinnitus in patients with temporomandibular disorder: a retrospective population-based cohort study. Eur Arch Otorhinolaryngol. 2016;273(1):203–8. 10.1007/s00405-015-3491-2 . 25573837

[53] Ralli M, Greco A, Cialente F, Stadio AD, Longo L, Ciofalo A, et al Somatic Tinnitus. Int Tinnitus J. 2017;21(2):112–21. Epub 2018/01/18. 10.5935/0946-5448.20170022 . 29336129

[54] Weisz N, Hartmann T, Dohrmann K, Schlee W, Norena A. High-frequency tinnitus without hearing loss does not mean absence of deafferentation. Hear Res. 2006;222(1–2):108–14. Epub 2006/11/03. 10.1016/j.heares.2006.09.003 . 17079102

[55] Schaette R, McAlpine D. Tinnitus with a normal audiogram: physiological evidence for hidden hearing loss and computational model. J Neurosci. 2011;31(38):13452–7. Epub 2011/09/24. 10.1523/JNEUROSCI.2156-11.2011 . 21940438 PMC6623281

